# Supplementary material for: Are carbon emissions trading and green financial instruments synergistic? -Comprehensive quantitative research based on content analysis
Source: PLoS One. 2024 Mar 7;19(3):e0298601. doi: 10.1371/journal.pone.0298601 (PMC10919601; doi:10.1371/journal.pone.0298601)
Supplement: S1 Data — (ZIP) [file pone.0298601.s001.zip › Data/地级市Z_F工作报告.zip!/╣π╬≈/▒▒║ú╩╨/└┤╫╘╣1⁄2╓┌║┼í╛┬φ┐╦╩2╛▌═°í┐_2012.docx]

各位代表：

现在，我代表北海市人民政府向大会作政府工作报告，请予审议，并请市政协委员和其他列席人员提出意见。

一、2011年工作回顾

2011年是极不平凡的一年。在自治区党委、政府和市委的正确领导下，全市人民深入贯彻落实科学发展观，坚持“真抓实干、把事干成、造福百姓”的理念，奋力推进北海三年跨越发展工程，实现了“两年明显见成效”目标和“十二五”发展良好开局，推动北海进入了产业带动跨越发展新阶段。

——经济发展实现新突破。完成地区生产总值496.6亿元、增长18.2%，规模以上工业产值550.6亿元、增长71.9%，全社会固定资产投资603.1亿元、增长26.1%，财政收入57.54亿元、增长22.16%，完成数分别相当于2008年的1.8、2.9、3.0和2.1倍。2009年至2011年，经济增长速度分别高出全区2.3、3.4和5.2个百分点，分别高出全国7.1、7.2和9个百分点，增速逐年加快，综合经济实力不断增强。

——石化大项目建成投产。经过18个月的紧张建设，北海炼油异地改造石化(20万吨/年聚丙烯)项目如期建成投产，北海人民祈盼多年并为之不懈努力的石化大项目终于梦想成真。预计项目今年税收达38亿元，拉动广西财政收入增长2个百分点。

——电子信息产业爆发式增长。完成产值300亿元、增长60.4%，提前一年实现北海三年跨越发展工程目标，电子信息制造业产值占全区60%以上。冠捷科技、国钰电子、建准电子项目建成投产，朗科国际存储科技产业园、三诺高新科技园开工建设。

——诚德新材料项目投产见效。一期工程投产首年完成产值74.7亿元，占全市规模以上工业总产值的13.6%，拉动全市工业增长23.3个百分点。二期工程即将投产，三期工程正加紧推进。

——承办世界客属第24届恳亲大会喜获圆满成功。经过长达三年的精心准备，我市成功办成一届规模大、规格高、影响广的世客会，得到了广泛好评，提升了北海的知名度和美誉度，促进了开放合作和经济社会发展。

——保障和改善民生工作成效显著。大力建设民生路网工程三期项目，投资3.77亿元改造和修建13条道路，目前已基本完工，方便了市民出行，提升了城市形象；实施千里乡村公路畅通工程，2011年安排的149个项目全部完成。市人民医院、中医院和铁山港区人民医院综合住院大楼投入使用。实现社会消费品零售总额127.29亿元、增长18%，城镇居民人均可支配收入18656元、增长11.1%，农民人均纯收入6248元、增长15%，居民消费价格涨幅控制在5.5%、低于全区0.4个百分点。

一年来，我们主要做了以下工作：

一是着力增强城市发展的产业支撑。按照定人员、定职责、定时间、定进度的“四定”办法，强力推进280个重点项目，完成投资254.6亿元，项目开工率、竣工率和完成投资额均高于上年。大力引进电子龙头企业，电子信息产业发展势头迅猛。320万方原油商业储备基地工程（首期80万方）、湛江至铁山港原油管道工程投入试运行，北海至南宁成品油管道工程试输水成功。推动园区经济持续高速增长，完成规模以上工业产值440.9亿元、增长75.7%，占全市规模以上工业产值的八成以上。北海工业园区完成产值170亿元，增长76.1%；北海出口加工区完成产值108.3亿元、增长50.3%，成为全市第二个产值超百亿元的园区；铁山港工业区完成产值95.6亿元，增长5.9倍；高新技术产业园区获批为国家级科技企业孵化器。启动三大千亿元产业发展规划和园区控制性详细规划编制工作。全市完成规模以上工业增加值135.8亿元，增长47.1%。

二是着力推进社会主义新农村建设。坚持统筹城乡发展，全面落实各项强农惠农政策，兑现各项支农补贴7.92亿元，各级财政投入农林水资金增长114.2%。大力发展优势特色农业、高效设施农业、休闲观光农业，高效设施农业面积达12万亩。林浆纸原料林基地建设取得新进展，发展林下经济的成功经验被国家及自治区总结推广。加大农村基础设施建设力度，完成水利投资7.6亿元，解决5.01万人饮水安全问题，农村自来水普及率提高到61%。成功抵御第17号强台风“纳沙”袭击，积极抓好抗旱工作。全市完成农林牧渔业总产值183.9亿元，增长3.1%。

三是着力发展以旅游为龙头的现代服务业。接待国内游客1100.79万人次、增长17.3%，实现国内旅游收入86.07亿元、增长28.14%。启动修编《北海市旅游产业发展总体规划》，加快实施涠洲岛旅游区发展规划、总体规划和控制性详细规划，启动建设涠洲岛旅游区首批10个基础设施项目。建成冠岭山庄、金昌开元名都酒店和银滩一号会展中心，开工建设冠岭国际会议中心。投入7500万元专项扶持资金补贴航空航线发展，新增和恢复7条航线，航线达到17条、通达城市18个。完成港口货物吞吐量1590.44万吨，增长27.18%。

四是着力深化改革开放。完成水产供销总公司、化肥厂、淀粉工业总厂3家规模以上企业改制工作。集体林权制度主体改革通过自治区检查验收。积极发挥国家历史文化名城优势，启动与广州、泉州等6城市联合申报“海上丝绸之路”世界文化遗产工作，大型历史舞剧《碧海丝路》赴马来西亚、斯里兰卡演出受到广泛好评。积极推进桂台合作，合浦县公馆镇与花莲县寿丰乡签署交流合作协议。大力推进口岸扩大开放，同时配合重大项目建设做好口岸临时开放工作。新引进项目130个，实际到位资金310亿元、增长25%，实际利用外资2.1亿美元。获批为首批国家外贸转型升级专业型示范基地（水产品）。完成进出口总额17.1亿美元、增长25%，高新技术产品、电子信息产品、加工贸易产品进出口总额等多项指标居全区首位。

五是着力推进生态宜居文明城市建设。不断优化城市发展布局，推动北海组团和铁山港（龙潭）组团互动发展。加快推进钦北铁路扩能改造、玉铁铁路、玉铁高速公路和铁山港公用码头3号、4号泊位建设，建成银滩三号路、新世纪大道等城市主干道。实施城乡风貌改造和农村危房改造工程，推进红坎污水处理厂二级处理二期等项目前期工作，完成广东南路、云南南路等一批污水管网工程。北郊水厂建成投产，结束了我市以地下水为单一原水的供水历史。深入实施净化、绿化、彩化、亮化、美化工程，大力开展人居生态环境建设，成功获批为国家园林城市，建成区绿化覆盖率达37.9%。提高城市规划、建设和管理水平，成为广西首个“全国数字城市建设示范市”。积极创建国家环境保护模范城市和节水型城市，环境空气质量优良率保持在全国前列，超额完成节能减排年度任务。

六是着力保障和改善民生。投入民生领域资金80.3亿元，增长35%。基本建成合浦汉文化公园主体工程，建成20个村级公共服务中心，在全区率先实现群众艺术馆、图书馆、少儿图书馆免费开放。加快推进北海中学迁建项目，妥善解决进城务工人员子女义务教育问题，职教攻坚通过自治区验收，高校在校生规模超过2.9万人；建立从基础教育到高等教育的资助体系，资助学生4.33万人次、金额达3228.6万元。大幅提高城镇医疗保险基金最高支付限额，加快普及“平价药”，公办基层医疗机构实现国家基本药物零差率销售。进一步加强食品药品安全监管。新型农村合作医疗参合率居全区首位，筹资标准提高到每人每年230元，银海区新型农村社会养老保险试点参保率达89.1%。城市和农村低保实现应保尽保。深入开展全民健身活动，参加第十二届区运会取得历史最好成绩。人口计生工作取得新成效，圆满完成自治区下达的任务。城镇新增就业人数4.53万人、城镇登记失业率3.05%，农村劳动力转移就业新增2.06万人。积极开展法律援助，受理法律援助申请747件。加大廉租住房、经济适用房、限价商品住房、公共租赁住房建设和棚户区改造力度，超额完成保障性住房年度建设任务。年初提出的12件为民办实事项目基本完成。采取投放“平价米”、“平价油”、“平价肉”等措施，稳定居民消费价格总水平，有效保障低收入群体基本生活。

一年来，我们切实加强政府自身建设，创新社会管理理念，发挥政府社会管理主导作用。市政务服务中心高效规范运作，投资者和群众满意率达95.69%。在13个乡镇、街道建成政务服务中心，初步形成市、县（区）、乡镇（街道）三级政务服务体系。扎实推进政务公开和政府信息公开，合浦县被列为全国加强县级政府政务公开和政务服务试点县。大力优化发展环境，在全国250个地级市城市商业信用环境评比中名列前三位，入选中国最具投资潜力城市50强；民意调查显示，群众对我市“政府办事公开的评价”满意率居全区首位。全面落实党风廉政建设责任制，不断完善反腐倡廉制度，严格遵守各项换届纪律。积极探索公共资源交易监管新模式，在全区率先开展电子招投标试点。创新应急管理体制机制，加强队伍建设，强化应急演练，有效处置突发公共事件。全面启动“六五”普法，积极化解各种矛盾，扎实推进平安北海建设，推动安全生产和社会治安形势持续好转，努力消除影响社会和谐稳定的因素，人民群众的安全感不断增强。

一年来，我们切实加强民主法制建设，自觉接受市人大及其常委会监督，认真执行各项决议、决定，及时报告工作；主动加强与市政协联系，积极支持政协履行参政议政职能。坚持邀请人大代表、政协委员列席市政府常务会议。切实加强人大代表建议和政协提案办理工作，办结率和满意率均达100%。广泛听取各民主党派、工商联及无党派人士意见，支持工会、共青团、妇联等人民团体更好地发挥作用。

各位代表!成绩来之不易。这是自治区党委、政府和市委正确领导、科学决策的结果，是市人大、市政协有效监督和大力支持的结果，是历届班子历任领导打下良好基础的结果，是广大干部群众团结拼搏的结果。在此，我谨代表市人民政府，向全市人民特别是给予政府工作大力支持的人大代表、政协委员、离退休老同志及各民主党派、工商联、人民团体、无党派人士，向驻市人民解放军和武警部队官兵，向中央、自治区驻市单位，向所有关心、参与和支持北海建设的海内外朋友，表示崇高的敬意和衷心的感谢！

我们清醒地认识到，当前北海经济社会发展中还存在不少困难和问题：用地、用海、审批核准等因素在一定程度上制约了项目建设，保持投资较快增长难度较大；受用电紧张、原材料价格上涨、用工成本上升、融资困难等因素影响，部分企业经营困难；物流瓶颈问题仍然突出，口岸开放未能满足优势产业发展的迫切需要；农业增产、农民增收难度较大，统筹城乡协调发展任务比较艰巨；保障和改善民生还有很多工作要做，特别是涨价因素较多，保持物价总水平基本稳定难度加大；城市建设管理还存在不少薄弱环节。个别政府部门和少数工作人员作风还不够实、效率还不够高、执行力还不够强。对于这些困难和问题，我们一定高度重视，采取更加有力的措施加以解决。

二、2012年工作目标和主要任务

今年是实施北海三年跨越发展工程的决战之年，也是实施“十二五”规划承上启下的关键一年。今年政府工作的总体要求是：以邓小平理论和“三个代表”重要思想为指导，深入贯彻科学发展观，认真落实自治区党委关于加快实现富民强桂新跨越的战略决策，按照市第十次党代会以及市委十届二次全会的决策部署，以科学发展为主题，以加快转变经济发展方式为主线，认真落实《国务院关于进一步促进广西经济社会发展的若干意见》和《广西北部湾经济区发展规划》，坚持“真抓实干、把事干成、造福百姓”的理念，按照“稳中求进、好中求快”和“稳增长、控物价、调结构、惠民生、抓改革、促和谐”的要求，大力发展优势产业、特色农业和现代服务业，全面加强城市建设管理，着力保障和改善民生、加强和创新社会管理，奋力夺取北海三年跨越发展工程的全面胜利，把北海基本建设成为区域性国际化的现代产业集聚基地、旅游商贸物流中心、开放合作重要平台和生态宜居文明城市。

2012年全市经济社会发展的主要预期目标为：生产总值增长20%以上、达到600亿元；财政收入增长73.79%、达到100亿元；全社会固定资产投资增长20%、达到725亿元；规模以上工业总产值增长80%、达到1000亿元；实际利用外资2.5亿美元；外贸进出口总额(含保税物流货值)38亿美元；社会消费品零售总额增长27.27%、达到162亿元；城镇居民人均可支配收入增长11%、达到2万元；农民人均纯收入增长12%、达到7000元；居民消费价格指数控制在4%左右；全面完成自治区下达的节能减排任务。

上述目标实现后，北海的人均地区生产总值将达到全国平均水平，人均财政收入将进入全区先进行列，工业化进程将进入加速发展阶段，北海的综合实力将迈上一个新台阶，持续繁荣发展的产业支撑将更加稳固，将为广西北部湾经济区加快建设“三基地一中心”作出更大的贡献。

夺取北海三年跨越发展工程的全面胜利，开创北海科学发展的新局面，是全市人民的殷切期盼，是时代发展的必然要求，是我们肩负的光荣使命。尽管面临不少困难和挑战，我们一定坚定信心、迎难而上，一定真抓实干、勇挑重担，一定把事干成、不辱使命!

我们将主要抓好以下重点工作：

(一)大力发展优势产业，加快形成产业集群和产业基地

推动电子信息、石油化工、临港新材料等优势产业扩大规模、延伸产业链，积极改造提升特色传统产业，加快发展战略性新兴产业，全面发展海洋经济，大力发展非公有制经济，进一步夯实城市发展的产业支撑。

加快建设千亿元电子信息产业基地。按照“一园多区”模式，统筹推进各园区电子信息产业发展。推动冠捷科技、建兴电子、德昌电机等企业尽快达产或扩大产能，朗科、三诺、六禾二期、景光三期、国钰二期等项目早日建成投产。继续引进一批大型电子企业和关联配套企业，提升产业配套能力和市场竞争力。加快软件开发、集成电路、信息服务等相关行业发展。力争实现电子信息产业产值450亿元。

加快建设千亿元石油化工产业基地。抓好铁路、码头、仓储等基础设施建设，抓紧完成项目卫生防护区征地搬迁工作，建成北海原油商业储备基地二期项目，推动北海炼油异地改造石油化工（20万吨/年聚丙烯）项目满负荷运行。抓紧做好千万吨级炼化一体化项目前期工作，推进广西LNG项目建设，建成玉柴润滑油三期扩建改造项目。围绕石化下游产品开发，推进和源油浆、中石化配套检维修等项目建设，加快上海嘉盛、广西强强碳素等项目前期工作。实现石油化工产业产值300亿元以上。

加快建设千亿元临港新材料产业基地。建成投产诚德新材料项目二期、三期工程，推进矿渣微粉项目、固溶线项目，力争开工建设四期120万吨/年冷轧板项目。争取在我市召开全国不锈钢年会，积极引进一批下游生产企业。竣工投产渤海农业高蛋白饲料项目，开工建设台玻特种玻璃生产项目。实现临港产业产值300亿元以上。

加快发展其他优势产业。着力打造北部湾能源基地，开工建设神华国华广投北海能源基地、北海电厂二期工程，加快推进6个110千伏至220千伏送变电工程。改造提升水产品加工业、果蔬加工业，力争实现农产品加工业产值150亿元。力争林浆纸一体化项目早日开工，加快华润水泥等项目建设。促进修造船、建材、机械等传统产业转型升级，积极发展先进装备制造、海洋生物和新能源产业。

强化工业运行保障。大力争取自治区用电指标支持，加强科学调度，鼓励企业自备电源发电，积极解决企业“用电难”问题。加大公共就业服务力度，完善产业园区生活配套设施，积极解决企业“用工难”问题。完成千亿元产业发展规划编制工作，加快千亿元产业园规划建设，力争北海工业园区升级为国家级经济技术开发区，推进高新技术产业园区软件与集成电路产业园、铁山港东岸临海产业园建设，推动合浦工业园区扩容。实现园区工业总产值突破800亿元，力争铁山港工业区超过500亿元、北海工业园区超过200亿元、出口加工区超过150亿元。

实施“抓大壮小扶微”工程。落实扶持政策，推动企业做大做强，力争新增年销售额100亿元以上企业2家；新增10亿元以上企业3家，总量达到18家；新增亿元以上企业6家，总量达到66家；新增上规模企业15家，总量达到172家；扶持发展微型企业400家以上。推动工业化和信息化深度融合，新增信息化应用企业15家、信息化示范企业2家。积极支持企业上市直接融资。完成更新改造投资291亿元。

(二)强力推进项目建设，促进投资稳步增长

坚持“四定”办法，按照一个项目、一个工作组、一套工作方案的“三个一”要求，制订完善项目会商、约谈、奖励制度，全力抓好重大项目建设，带动推进其他中小项目，发挥投资的强大拉力。实施重大项目301项、总投资2048亿元，年内完成投资277亿元。

农林水设施方面。完成投资3.3亿元以上。开工建设七星围和大坎海堤、农村饮水安全工程、营盘中心渔港等项目，力争开工建设北部湾滨海植物园、百曲围海堤、乾江围海堤，加快建设古城海堤、市蔬菜配送中心二期等项目，建成国家现代农业示范区旱涝保收基本农田项目、合浦水库灌区2011年度节水改造工程。

旅游设施方面。完成投资21亿元以上。开工建设涠洲岛环岛路、五彩滩景区等项目，加快建设北海老城保护改造、中信国安生态旅游区、冠岭国际会议中心、富丽华改造等项目。

交通基础设施方面。完成投资65亿元以上。加快推进玉铁铁路、铁山港铁路支线，完成钦北铁路扩能改造、中石化项目专用线，抓紧完成铁山港站至广投配煤中心专用线、港口铁路专用线前期工作。开工建设北铁一级公路改造等项目，加快建设贵港至合浦高速公路、加快广西滨海公路(大风江至高德段)前期工作，建成玉铁高速公路(北海段)、营盘经石头埠至闸口二级公路C段等项目。开工建设铁山港公用码头5至8号泊位，加快建设石步岭港区三期工程、北海邮轮码头，建成铁山港公用码头3号和4号泊位。

园区基础设施建设方面。完成投资25亿元以上。开工建设出口加工区B区及配套产业园区基础设施项目、铁山港口岸联检综合服务中心、铁山港石化产业园区配套路网、铁山港工业区经四路、三号路A段和八号路B段，加快建设铁山港工业区七号路西段、台湾电子产业园基础设施项目、铁山港东岸龙腾路和榄根路，建成中石化项目配套道路、铁山港工业区排水明渠(一期)，加快铁山港航道疏浚二期扩建等项目前期工作。

社会事业方面。完成投资6亿元以上。开工建设市十中、海城区十八小、银海区一小、市体育馆、市综合健身馆、市群艺馆迁建、平安北海二期等项目，加快建设北海中学异地搬迁一期、北部湾体育中心一期、山东省体育局北海训练基地、第二人民医院迁建、银海区人民医院、合浦精神病医院等项目，建成海城区十七小、市武警支队指挥中心和消防指挥中心，加快新建市图书馆、青少年宫项目前期工作。

(三)大力发展现代特色农业，扎实推进社会主义新农村建设

坚持在工业化、城镇化深入发展中同步推进农业现代化，落实各项强农惠农政策，大力促进科技兴农，切实加强“三农”工作，加快形成城乡经济社会发展一体化新格局。

大力发展现代特色农业。严格保护基本农田，提高粮食综合生产能力，确保全年粮食播种面积达120.5万亩、总产量36.5万吨以上。推进农业产业化，着力打造水产百亿元产业和粮油、蔬菜、水果、蔗糖、畜禽、农业休闲观光等十亿元产业。认真落实“菜篮子”责任制，实施“放心粮油工程”、“农户科学储粮工程”，增强农产品供给保障能力。加快发展高效设施农业，打造一批亩产值超万元的“万元片区”、人均纯收入1万元以上的“万元村屯”和年纯收入10万元以上的“致富示范农户”。建设改造一批乡镇农产品贸易市场，培育村级农贸市场，发展“一村一品”经济强村。加快发展生态农业和创意农业。大力发展林业和林下经济，建设乌家林下经济走廊。加快发展畜牧业，扶持标准化规模养殖场发展。巩固发展水产养殖业，积极拓展远洋渔业。提升农业技术推广能力,大力发展农业社会化服务。加快建设北海国家农业科技园区、合浦县国家现代农业示范区。

大力改善农村基础设施。加快水利改革发展，推进病险水库除险加固、海河堤防建设、中小河流治理、灌区节水改造工程建设。推进千里乡村畅通工程，建设通行政村硬底化公路173公里、通自然村硬底化公路124公里，惠及农村人口23.7万人。加快北海内港、营盘、沙田等渔港基础设施建设，实施库区、移民安置区基础设施和移民新村建设工程。加快实施规模畜禽场沼气治理和农村户用沼气工程，推广高效种养生态循环模式。抓好农村危旧房改造、环境整治、污水排放、垃圾处理和电网改造。推广农业防灾减灾技术，提高抵御自然灾害能力。

坚持统筹城乡发展。提高农村义务教育质量和均衡发展水平，加快农村中等职业教育免费进程。健全农村三级医疗卫生服务网络，提升基层医疗服务能力。巩固推进广播电视“村村通”和农村电影放映，基本建成农村公共文化服务体系。落实“连片开发、整村推进、扶贫到户”的要求，开展新一轮扶贫开发攻坚，确保贫困村农民人均纯收入增速达到全市平均水平1.1倍以上，年内有12%以上的贫困人口实现脱贫，重点抓好39个贫困村的扶贫开发。力争经过几年的努力，在全区率先消除绝对贫困。

(四)做大做强旅游产业，促进现代服务业繁荣发展

促进服务业与工业化、城镇化融合互动发展，不断提高现代服务业发展水平。实现接待国内游客1300万人次，国内旅游收入突破100亿元。

全面提升旅游业。抓好涠洲岛旅游区基础设施建设，加快推进首批10个项目，启动中心片区等第二批项目，全面加快整体开发步伐。促进银滩景区转型升级，加快打造具有浓郁滨海特色的综合型高品质旅游度假区。推进金海湾、山口红树林、星岛湖等旅游资源富集区域开发，抓好涠洲岛鳄鱼山景区创5A和北海老城、金海湾红树林生态休闲旅游区创4A等景区标准化建设。精心培育特色文化精品旅游线路。新建成高星级酒店1家以上。加大航空市场培育力度，力争新开通航线2-3条，通达城市20个。推动北海至越南下龙湾海上旅游航线健康发展。加强旅游宣传营销和市场监管，举办旅游文化节庆活动，积极引进大型旅游企业，全面提升竞争力。

加快发展商贸物流业。制定实施商业网点规划和大型专业市场发展规划，推进大型专业市场建设，规划建设商业步行街，争取引进一批高端品牌连锁店。开工建设出口加工区国际名品展示中心。加强物流体系建设，培育物流海运航线和陆运专线，优化通关服务，努力降低企业物流成本。积极引进大型物流企业，推进港口与产业园区联动发展。

积极发展其他服务业。保持房地产投资适当规模，加强市场管理，促进房地产业平稳健康有序发展。推进“引金入北”，拓展政银企战略合作，引导金融机构调整信贷结构，扩大信贷规模，加强金融服务，稳健发展小额贷款公司，规范发展融资性担保机构。培育会展主体，开拓会展市场，发展特色会展。大力发展服务外包、技术研发、信息咨询、社区家政等面向生产和民生的服务业。

(五)扎实开展“城市建设管理年”活动，加快建设生态宜居文明城市

按照建设特大城市和生态宜居文明城市的要求，坚持高起点规划、高标准建设和人性化管理，全面抓好新城区建设、旧城区改造和长效机制管理，完善城市综合功能，提高城市宜居水平，优化城市整体形象。

突出规划龙头作用。加强城市发展战略和城市特色研究，提高城市规划管理科学化水平。完成《北海城市发展战略规划》，修编城市总体规划和近期建设规划，完善控制性详细规划。实施历史文化名城保护管理规定，构建历史文化名城保护体系。强化规划管理，对新建区域实施规划控制，对建成区实施功能改造，对建筑风格、外立面设计进行严格控制，严厉打击违法建设特别是抢建、抢种行为，维护规划的权威性和严肃性。加强土地储备，积极稳妥推进城市闲置土地处置。

加快新城区开发建设。坚持区城共建，逐步拓展城市空间，增强城市发展承载力。重点抓好廉州湾沿岸约10平方公里新城的开发，借鉴上海浦东、南宁琅东的开发经验，编制廉州湾总体规划，实施区域范围内岸线整治，完善基础设施和公用设施配套，规划建设现代化建筑群，打造集商务、金融、办公、生态居住和公益设施为一体，富有滨海特色和生命力、展示北海新形象的现代城市商务区。规划建设银滩商业步行广场，开工建设银滩大道、银滩二号路，加快银滩中区区域改造建设。完善银滩东区基础设施，力争年内启动开发建设。推进铁山港工业区港、区、城共建，加快建设滨江新区。推进合浦县城与市区一体化发展，扩大合浦县城规模。加快完善市区“七纵七横”主干路网，建成成都北路、南京路等次干道。加快小城镇建设，重点抓好山口、西场、福成、南康、营盘、涠洲等小城镇建设。坚持配套公用设施与新城区同规划、同建设，确保建成一片、完善一片。

改造提升旧城区。按照滨海城市的标准和规范，改造旧城区主要街道和小街小巷，完善供水、排水、排污、红绿灯、人行道、路灯、垃圾收集等设施，依法保护和合理改造历史文化街区，改善人居环境，努力打造特色突出、设施完善、环境优美的中心城区。加快环卫基础设施建设和环卫设备更新，新建一批垃圾中转站，新建、改造公共厕所20座。开展北部湾广场、侨港、外沙岛、老街等片区风貌整治提升工程。按照合理的服务半径范围，进一步完善旧城区教育、医疗卫生、农贸市场等功能设施配套。

提高城市综合管理水平。在全面集中整治、确保尽快取得明显成效的基础上，进一步加强和规范各项管理，强化综合执法，形成和完善长效机制。坚持推进“城乡清洁工程”，切实抓好道路保洁、垃圾收集清运、施工工地、户外广告、渔港码头、废旧品回收、道路桥梁管护和营运车辆的管理，大力开展臭水污染、乱摆乱卖、占道经营、运输车辆洒漏污染、违章建筑、乱挖虾塘等专项整治。加强交通管理，着力解决上下班高峰期部分路段交通拥堵问题。积极创新体制机制，提高信息化水平，形成分工明确、责权统一、保障有力、运转高效的城市管理新格局。加强宣传教育，提升市民素质，提高城市文明水平。

加强环境保护和生态建设。加快创建国家环境保护模范城市、节水型城市和国家级生态城市，争创中国人居环境奖。加强海域环境和农村环境整治，加大重要生态功能保护区和饮用水源地保护力度，启动涠洲岛珊瑚礁海洋公园申报建设工作。推进城市净化、绿化、彩化、亮化、美化，抓好新建公园建设，加强城市中心区、城市道路、单位庭院和住宅小区绿化。开工建设红坎污水处理厂二级处理二期、内涝整治二期、污泥处置中心和铁路以南10个污水管网项目，加快产业园区环保配套设施建设。扎实推进节能减排工作，严把建设项目环评审批关，加强清洁生产审核，淘汰落后产能。深入开展环保专项行动，加大环保执法和挂牌督办、责任追究的力度。

(六)深化重点领域改革，扩大开放合作

北海最大的优势在于开放，要通过开放促进改革，通过开放推动创新，更好地集聚生产要素，不断增创发展新优势。实现招商引资实际到位资金345亿元。

推进重点领域改革。深化财政各项改革，完善公共财政管理体制，健全财力与事权相匹配的财税体制，调动城区和园区发展经济的积极性。加强投融资平台建设与统筹管理、监管，建立“借用管还”良性机制。继续优化金融环境，积极防范金融风险。加快完成国有企业改制工作。抓好农村小额信贷、农业保险等综合改革。推进农村户籍管理、社会保障、集体林权制度配套等改革创新，依法促进农村土地承包经营权有序流转。做好科技、教育、医药卫生体制改革和事业单位分类改革。扎实推进扩权强县工作。

提升开放合作水平。完善口岸基础设施，力争实现北海口岸扩大开放。加强项目策划和储备，完善奖励激励和考核机制，充分调动各方面积极性，进一步提高招商引资成效。创新政府、企业和民间组织相互间的合作机制，推进以泛北地区为重点的国际区域合作，加强与珠三角、长三角、西南地区、港澳台地区的交流合作，逐步形成多层次、多元化的开放合作新格局。大力发展外向型经济，积极承接产业转移。加快国家科技兴贸创新基地、国家加工贸易梯度转移重点承接地、国家外贸转型升级专业型示范基地建设。加强外事侨务和台湾事务工作。

(七)推动文化大发展大繁荣，全面发展社会事业

加强社会主义核心价值体系建设。广泛开展理想信念、国情区情市情和形势政策教育，弘扬社会主义道德风尚。深入开展思想道德主题实践活动，净化社会文化环境。启动全国文明城市创建工作，深入开展群众性精神文明创建活动，加快提升社会文明程度。深入开展双拥活动，加强军政、军民团结。

大力发展文化事业和文化产业。深化文化体制改革，进一步兴起文化建设新高潮。加强历史文化遗产挖掘、保护和展示、利用，推进联合申报“海上丝绸之路”世界文化遗产工作，提升国家历史文化名城的影响力。繁荣文艺创作，深入实施文化精品工程。加强文化基础设施建设，推进文化惠民，深入开展“文化、科技、卫生”下乡活动，繁荣发展广场文化、社区文化、村镇文化、企业文化、校园文化。加快文化产业发展，引进、培育一批文化骨干企业，扶持发展动漫、创意、影视、文博等产业。加强文化市场管理。促进文化与旅游、科技融合，推动文化精品走向市场，扩大文化对外交流。

加快发展科技、教育、卫生、体育等社会事业。扎实推进科技创新体系建设，加大高新技术企业培育力度。优化教育资源配置，大力发展各类教育，力争分别引进两所全国著名的中学和高校，启动北海职业学院搬迁，开工建设一批中小学和幼儿园，争取用3年时间解决市区中小学布局不合理、班额过大的问题，加强校车安全管理。深化医药卫生体制改革，深入实施国家基本药物制度，提高公共卫生服务均等化水平，加快卫生基础设施建设，增强公共卫生事件应急处置和疾病预防控制能力。力争引进两所全国著名的医院，提升北海医疗服务水平。加大体育场馆建设力度，广泛开展全民健身活动，不断提高体育竞技水平。强化人口和计划生育目标管理责任制，推进人口信息化管理建设，继续稳定低生育水平。建立完善人才激励体系，加快集聚各类急需紧缺人才特别是高层次人才。积极推进残疾人社会保障体系和服务体系建设。重视发挥离退休干部作用。加快发展审计、统计、气象、海防、档案等其他事业。

(八)加强和创新社会管理，更好地保障和改善民生

提高社会保障水平。加快建设覆盖城乡居民的社会保障体系和社会救助体系，健全城乡居民低保制度，逐步提高保障标准。重点做好被征地农民的社会保障工作。发展社会福利和养老事业，做好老龄工作。积极稳控物价，完善社会救助和保障标准与物价上涨挂钩的联动机制，切实保障低收入群体基本生活。

加强就业创业工作。整合现有资源，打造“北部湾人力资源市场”，创建高水平就业指导和服务平台。引导和鼓励高校毕业生面向企业、基层和农村就业创业，突出抓好就业困难人员、退役军人、城镇失业人员、水库移民、失地农民和转产转业渔民等重点群体就业工作。努力构建和谐劳动关系，切实维护劳动者合法权益。实现农村劳动力转移就业1.5万人以上，城镇登记失业率控制在4%以内。优化创业环境，实施全民创业工程，支持农民工返乡创业。

切实提高社会管理水平。强化社会管理服务职能，充分发挥各级政府在社会管理中的主导作用。建立调处化解矛盾纠纷综合平台，完善大调解工作体系，努力从源头上预防和减少社会矛盾，畅通信访渠道，引导群众正确行使权利和履行义务。推进“六五”普法教育。提高社区、村居组织管理和服务辖区能力，充分发挥基层组织在社会管理中的骨干作用。健全社会治安防控体系，强化社会治安综合治理，加快推进天网工程，依法严厉打击“两抢一盗”、传销等违法犯罪活动，增强人民群众安全感。着力做好流动人口和特殊人群管理服务工作。强化食品药品安全监管，进一步提高安全水平。深化对烟花爆竹、交通运输、渔港安全等重点行业领域安全专项治理，继续推动安全生产形势持续好转。建立完善应急管理机制，增强基层应急管理能力，启动综合应急指挥平台建设，有效应对和处置各类重大突发事件和公共危机。

大力实施为民办实事工程。筹措资金16.24亿元，办好以下11项工程：

1．社保惠民工程。筹措资金1.1亿元，实现城乡居民社会养老保险制度全覆盖。筹措资金8620.8万元，为全市2.4万城市低保对象、5.2万农村低保对象发放最低生活保障补助，月人均补助标准分别达165元和62元。筹措资金不少于170万元，新建10个五保村。筹措资金2000万元，开展就业培训惠民工程，实现城镇新增就业2.5万人以上。

2．卫生惠民工程。筹措资金3.19亿元，新型农村合作医疗筹资标准提高到每人每年290元，财政对新型农村合作医疗补助提高到每人240元。为城乡居民免费提供基本公共卫生服务，建设标准化的城市社区卫生服务中心。实现公办村卫生室国家基本药物制度全覆盖。深入实施防治艾滋病攻坚工程、母婴健康“一免二补”幸福工程和地中海贫血防治计划。筹措资金62万元，开展家庭过期药品回收，增设过期药品回收点26个。

3．教育惠民工程。筹措资金8033万元，新建公办幼儿园8所，改建扩建4所，改制15所乡镇中心校附设幼儿园为公办幼儿园，确保75%的乡镇至少有1所公办中心幼儿园，村级幼儿园（班）普及率达80%。筹措补助资金1900万元，资助2万名农村义务教育阶段家庭经济困难寄宿生生活费。筹措资金2000万元，维修改造校舍1.25万平方米。筹措资金600万元，资助全市4000名普通高中家庭经济特别困难的在校学生。

4．强基惠民工程。筹措资金3666万元，新增19个行政村通硬底化（水泥）公路、总长50.3公里，修建自然村（屯）水泥路20公里以上。筹措资金861.6万元，修建贫困地区村屯道路77条，总长84.91公里。筹措资金1300万元，改造涠洲岛北岸道路约7公里。筹措资金500万元，扩宽修建涠洲岛博物馆至天主教堂道路4.5公里。筹措资金3500万元，开工建设涠洲岛污水处理厂及配套管网一期工程。筹措资金1400万元，解决3.5万农村居民饮水安全问题。筹措资金350万元，建设水库移民新村14个。

5．安居惠民工程。筹措资金6.6亿元，开工建设保障性住房、棚户区改造住房5750套，新增发放廉租住房租赁补贴750户，竣工保障性住房和棚户区改造住房3700套。筹措资金4800万元，改造农村危房3000户。完善住房保障工作制度，切实解决中低收入家庭住房困难问题。

6．农补惠民工程。筹措资金1165万元，落实水稻、玉米等粮食农作物良种补贴面积96.5万亩。筹措资金1160万元，落实农机具购置补贴项目。筹措资金15万元，对基层供销社的20个农资、日用品农家店进行改造建设。

7．生态惠民工程。筹措资金1175万元，实施“绿满八桂”绿化造林工程。筹措资金69万元，实施森林生态效益补偿项目。筹措资金690万元，建设农村户用沼气池1550座。筹措资金140万元，实施53个村（屯）农村环境综合整治项目。

8．文体惠民工程。筹措资金2200万元，建设88个村级公共服务中心。筹措资金30万元，开展文化惠民演出100场次。筹措资金82万元，补贴放映农村公益电影4116场，实现每个行政村每月放映一场电影。筹措资金120万元，安装20条健身路径。

9．计生惠民工程。筹措资金144万元，对6000对以上新婚夫妇进行国家免费孕前优生健康检查。筹措资金116万元，建设58个村级人口计生健康服务室和新家庭文化书屋。筹措资金160万元，足额兑现计划生育奖励扶助金、独生子女保健费。筹措资金280万元，落实非财政拨款的企、事业单位实行计划生育人员退休后的政策待遇。

10．城建惠民工程。筹措资金230万元，建设长青公园Ⅱ类地震应急避难场所。筹措资金50万元，在市城区主要交通路口安装两套环境噪音自动监测系统。筹措资金600万元，新建2座垃圾压缩收集站。筹措资金485万元，实施北海工业园区生活垃圾压缩转运站工程。筹措资金2146万元，设置一批交通护栏、标志、标线，维护城市交通安全。筹措资金1000万元，集中解决20条小街小巷“路难走、灯不亮”的问题。

11．平安惠民工程。筹措资金1800万元，在市辖县区各乡镇共60个较大的自然村安装300个电子监控设备。力争用三年的时间，完成345个较大的自然村安装电子监控设备任务，实现全市行政村全覆盖。

三、努力提高政府自身建设水平

各位代表！夺取北海三年跨越发展工程的全面胜利，对政府建设提出了更高的要求。我们将坚持“真抓实干、把事干成、造福百姓”的理念，切实提高驾驭全局和复杂局面的能力，提高推动科学发展的能力，以新的面貌、新的形象、新的能力、新的作风，确保全面完成各项目标任务。

第一，进一步提高行政执行力。认真开展“解放思想、赶超跨越”大讨论活动，按照“公开、公平、公正，依法依规，不造成国有资产流失”的原则，大胆解放思想，积极开拓创新，敢于担当、善于作为、讲求实效。坚持党的领导，认真贯彻落实市委重大决策部署。坚持政府工作“一盘棋”思想，既要守土有责、各司其职，又要密切配合、加强协作，增强领导班子合力，激发干部队伍活力。深入推行“四定”办法，做到事有专管之人、人有明确之责、责有限定之期。健全重大决策部署执行情况检查和专项督查制度，强化行政效能电子监察，严格行政问责，确保政令畅通、令行禁止。

第二，进一步弘扬真抓实干作风。深入推进创先争优，努力营造更加浓厚的干事创业氛围，切实践行戒骄、戒懒、戒空、戒虚、戒假、戒奢，真干实干苦干。发扬“两脚泥”精神，注重调查研究，广泛了解社情民意，深入困难矛盾多、群众意见大、发展制约重的地方，做到民有所呼、政有所应，真心实意为群众解难题、办实事。创新行政审批服务方式，加强电子政务建设，健全重大项目“绿色通道”和联合审批常态化机制。完善市政务服务中心建设，加快打造“全国一流、广西最优”的政务服务中心。拓展政务服务领域，继续推动政务服务向基层延伸，提高服务群众、服务基层、服务企业的水平。

第三，进一步提高依法行政水平。坚持把依法行政贯穿到政府决策、执行和监督全过程。规范行政决策程序，促进依法决策、科学决策、民主决策。规范行政执法行为，落实行政执法责任制，做到规范执法、公正执法、文明执法。认真执行人大及其常委会决议、决定，自觉接受人大及其常委会的依法监督和政协的民主监督，重视做好人大代表建议和政协提案办理工作，认真听取民主党派、工商联、无党派人士和人民团体的意见，主动接受社会监督和舆论监督。深化政务公开和政府信息公开，健全政府信息发布制度，让人民群众更有效地监督政府。

第四，进一步推进政府廉政建设。严格执行党风廉政建设责任制，认真落实廉洁自律各项规定。强化领导干部经济责任审计。加强对重大项目的审计和监督，把项目建成优质工程、安全工程、阳光工程、廉洁工程。认真开展重点领域渎职侵权专项整治工作，严肃查处违法违纪行为，坚决纠正损害群众利益的不正之风，着力解决反腐倡廉建设中人民群众反映强烈的突出问题，以廉政建设的新成效惠之于民、取信于民。

各位代表!当前北海正处在大有可为的重要战略机遇期，蓝图鼓舞人心，形势催人奋进，任务艰巨繁重。让我们紧密团结在以胡锦涛同志为总书记的党中央周围，在自治区党委、政府和市委的正确领导下，不动摇、不懈怠、不折腾，以更加振奋的精神、更加开阔的视野、更加务实的作风，开拓创新，扎实工作，奋力夺取北海三年跨越发展工程的全面胜利，以优异成绩迎接党的十八大胜利召开！
